# The molecular mechanisms of incretin resistance in Type 2 Diabetes Mellitus (T2DM)

**DOI:** 10.1038/s41387-026-00426-w

**Published:** 2026-05-06

**Authors:** Rawayh Muslim Albaghlany, Abbas Ali Mansour

**Affiliations:** 1 Department of Medical Microbiology, College of Dentistry, University of Basrah, Basrah, Iraq; 2 Faiha Specialized Diabetes, Endocrine, and Metabolism Center (FDEMC), University of Basrah, Basrah, Iraq

**Keywords:** Cell biology, Endocrinology

## Abstract

Incretin hormones, specifically glucagon-like peptide-1 (GLP-1) and glucose-dependent insulinotropic polypeptide (GIP), serve as crucial mediators of postprandial glucose homeostasis by primarily enhancing glucose-stimulated insulin secretion. Research indicates that the incretin effect accounts for approximately 50% of insulin secretion in individuals without diabetes, which is significantly reduced to 30% or less in those with Type 2 Diabetes Mellitus (T2DM). The mechanisms underlying this incretin resistance have emerged as critical causes of postprandial hyperglycemia. Incretin-based therapies, including GLP-1 receptor agonists (GLP-1RAs) and DPP-4 inhibitors, have demonstrated efficacy in managing T2DM; however, intrinsic resistance mechanisms may limit their effectiveness. Understanding the processes by which T2DM affects incretin action, from hormone secretion to the modulation of signal transduction, is essential for optimizing current therapies and developing new interventions to enhance β-cell responsiveness and improve glycemic control. The concept of incretin resistance has a well-established history in literature, dating back to at least the early 1990 s. It is used to describe a reduced insulinotropic responsiveness to incretin hormones in individuals diagnosed with T2DM. This review examines how hyperglycemia, chronic inflammation, and genetic susceptibility collectively inhibit incretin signaling through distinct yet interconnected molecular pathways. This impairment exacerbates postprandial hyperglycemia and accelerates β-cell dysfunction. We propose novel hypotheses regarding selective β-arrestin signaling, enhancers, epigenetic regulation, interactions between gut microbiota and incretins, inflammation-induced endoplasmic reticulum (ER) stress, and genotype-specific therapeutic responses. The hypotheses presented in this review serve as a framework for future research and therapeutic development to combat the phenomenon of incretin resistance and improve the clinical management of T2DM.

## Introduction

GLP-1 and GIP are gut-derived hormones released in response to nutrient intake. Together, they represent ~50% of postprandial insulin secretion in healthy humans, an effect known as the incretin effect [1]. The incretin effect is significantly influenced by the glucose load; it is relatively modest, approximately 25%, following the administration of 25 g of oral glucose. In contrast, this effect can reach levels of 75–80% when a 100-g glucose load is administered to healthy individuals [2]. GLP-1, secreted by intestinal L-cells, and GIP, released from K-cells, activate G protein-coupled receptors (GPCRs) on the surface of the pancreatic β-cells, leading to a glucose-dependent insulin release. It helps the effective disposal of oral glucose and reduces excessive glycemic excursions [3]. GLP-1 also suppresses glucagon secretion and slows gastric emptying, and GIP has further roles in fat metabolism [4].

Individuals with T2DM exhibit a reduced incretin effect, which has led to the prompt development of incretin-based therapies [5]. Incretin activity was enhanced by introducing the GLP-1RAs and DPP-4 inhibitors, now widely employed for treating T2DM [6]. Given that GIP is limited in treating T2DM, pharmacological GLP-1RAs have improved glycemic control [7]. Intravenous GLP-1RAs also normalized T2DM plasma glucose levels, while the high dose of GIPRAs does not stimulate enough insulin secretion [8]. These data indicate selective incretin resistance, in particular to GIP, in subjects with T2DM. The loss of the incretin effect has been well documented in the literature since the early 1990s, making it an established concept in the pathophysiology of T2DM [9].

The condition of β-cell overload arises from prolonged stimulation of pancreatic β-cells by endogenous or exogenous secretagogues, resulting in a sustained high demand for insulin secretion. This condition is characterized by diminished first-phase insulin secretion, an altered proinsulin-to-insulin ratio indicative of impaired precursor processing, and the activation of cellular stress pathways, including the unfolded protein response (UPR), alongside markers of oxidative stress. Collectively, these alterations signify functional exhaustion and structural deterioration of β-cells, culminating in a gradual impairment of endocrine function and involving in the diminishing of the incretin effect [10].

We detail novel hypotheses and significant research frontiers relevant to molecular processes, including inflammation, epigenetic regulation, microbiota interactions, and genotype-dependent responses. A comprehensive understanding of these complex interactions is critical for developing targeted therapeutics, optimizing glycemic control, and enhancing overall clinical outcomes in patients with T2DM. This review presents a mechanistic framework that classifies the interactions among hyperglycemia, chronic inflammation, and genetic predisposition, which collectively disrupt incretin-mediated glucose homeostasis.

## Molecular mechanisms of incretin resistance

### Defective of incretin secretion in T2DM

#### GIP secretion

A deficiency in GIP secretion is rarely observed in patients with T2DM. Postprandial levels of GIP are found to be normal or only slightly reduced. Subsequent analysis utilizing specific assays demonstrated only a modest impairment of GIP release in T2DM, which was considered not clinically relevant. Therefore, the GIP defect in T2DM is not mainly due to decreased hormone release [11]. Hypersecretion of GIP is often observed with obesity, a common comorbidity of T2DM. Consequently, such deficits may remain unnoticed in subjects with T2DM due to an elevated GIP response to food in obese individuals, which can conceal the reduction of GIP secretory abnormalities [12].

#### GLP-1 secretion

The secretion of GLP-1 is impaired in individuals with T2DM, with this deficiency being more pronounced during the late postprandial period compared to the early postprandial period. Such an impaired late response can significantly affect glucose control [13]. The early postprandial phase begins about 10 to 15 min after food consumption. During this stage, the initial peak of GLP-1 enhances insulin secretion in response to rising glucose levels while suppressing glucagon release. This rapid signaling also involves early gastric emptying and feeding behavior, although these functions are more significantly influenced during the late postprandial phase [14]. The late postprandial phase typically occurs 30 to 60 min (or longer) after a meal and may last several hours. During this period, GLP-1 levels rise, essential for maintaining postprandial glucose homeostasis by providing sustained insulin action [15]. The late-phase action of GLP-1 plays a crucial role in preventing overeating and regulating caloric intake by slowing gastric emptying and enhancing the sensation of satiety [16]. Individuals with T2DM exhibit an attenuated incretin response following meal ingestion, insufficiently increasing GLP-1 levels. This deficit is more pronounced in patients in their advanced T2DM stage or poor glycemic control than in the earlier stage of individuals with T2DM [17].

Furthermore, the impaired GLP-1 secretion is a secondary defect of the diabetic phenotype, as non-diabetic twins and first-degree relatives of T2DM subjects exhibit normal GLP-1 secretion profiles. This suggests that the deficiency of GLP-1 secretion is secondary to the diabetic state rather than being genetically inherited. Instead, chronic hyperglycemia, insulin resistance, and obesity are linked as contributing factors to L-cell dysfunction, thereby limiting GLP-1 release [18]. Thus, incretin resistance in T2DM cannot be entirely attributed to inadequate hormone secretion; however, a mild GLP-1 secretory defect can be identified, particularly in the latest stages of T2DM progression compared to healthy subjects, which further diminishes the overall incretin effect [19]. There is substantial evidence supporting the existence of a gut microbiota-GLP-1 axis in preclinical models, highlighting the role of microbial metabolites, such as short-chain fatty acids (SCFAs), in stimulating GLP-1 secretion from L cells via FFAR2/FFAR3 receptor activation and bile acid -TGR5 agonism, as demonstrated in cellular and animal studies. However, in vivo human intervention studies present contradictory findings, with some research indicating that SCFA exposure does not significantly stimulate GLP-1 secretion [20, 21].

The comparative analysis of GIP and GLP-1 secretion in individuals with T2DM is presented in Table 1.

**Table 1:** Comparison between GIP and GLP-1 regarding release and action in T2DM.

Parameter	GIP	GLP-1
Basal Levels	In T2DM, the typical level is normal or slightly raised.	Generally, the basal levels are near-normal, though some studies have reported slight reductions.
Early Postprandial Secretion	Despite a rapid early-phase increase may still occur, the insulinotropic effect is reduced due to β-cell resistance.	The initial phase can be diminished compared to healthy controls, resulting in a reduced insulinotropic effect.
Late Postprandial Secretion	The main issue is β-cell unresponsiveness to GIP, not necessarily GIP secretion itself.	Often markedly reduced in T2DM, resulting in inadequate postprandial insulin secretion and suboptimal glycemic control.
Insulinotropic Effect	The downregulation of the GIPR on the β -cells and the post-receptor defects impair the insulinotropic effect of GIP.	Generally, GLP-1 is better preserved than GIP in T2DM, but its lower levels reduce its potential benefit.

The table compares key GIP and GLP-1 secretion characteristics and their effects on T2DM. Both incretins exhibit abnormal secretion kinetics. GIP levels are typically normal or mildly elevated, but its insulinotropic response is significantly impaired due to the β-cell downregulation of its receptor. In contrast, GLP-1 secretion may be reduced or delayed, leading to insufficient postprandial insulin secretion and poor glycemic control. Although GLP-1 remains functional, its lower concentration in T2DM reduces its overall insulinotropic effect.

#### β-cell dysfunction and the diminished incretin effects

In the absence of hormonal stimuli, diabetic β-cells exhibit several deficiencies including: (i) an inability to release first-phase insulin effectively [22]; (ii) various alterations, including elevated proinsulin/insulin ratios, which indicate stress within the secretory pathway [23]; (iii) activation of oxidative stress and endoplasmic reticulum (ER) stress signaling, correlated with increased activity of the UPR through the PERK–eIF2α, IRE1–XBP1, and ATF6 pathways [24]; and (iv) transcriptional modifications that impair stimulus-secretion coupling in β-cells associated with T2DM, specifically affecting the gene expression of PDX1 and MAFA [25]. These pathological changes limit the maximum insulinotropic effect achievable through incretin-mediated signaling. However, this defect is only partial, as GLP-1 also stimulates cAMP-dependent exocytosis via the EPAC2/PKA pathway and exerts effects beyond the pancreas, including the induction of gastric emptying and suppression of glucagon secretion. In contrast, the insulinotropic efficacy of GIP is significantly diminished in the context of chronic inflammation and β-cell hyperglycemia [26].

### Downregulation and desensitization of incretin receptors

#### GLP-1R and GIPR expression in T2DM state

Receptor defects have been identified in hyperglycemic models, such as rats undergoing 90% pancreatectomy, as well as in multiple datasets derived from human islets [27, 28]. However, data obtained from human subjects exhibit considerable variability, influenced by differences among donors and the extent of glycemic exposure. Several studies have reported decreased mRNA or protein levels of GLP-1R and GIPR; nonetheless, these findings have not been consistently replicated across different studies, particularly within subpopulations of islets from individuals with T2DM [29]. Therefore, while receptor downregulation may contribute to the observed phenomena, it cannot be solely attributed to this mechanism. Additionally, the upregulation of miR-204 directly leads to post-transcriptional suppression and impaired receptor trafficking when chronic ER stress and UPR pathways become activated [30, 31].

#### Chronic stimulation and homologous desensitization

**Fig. 1:**
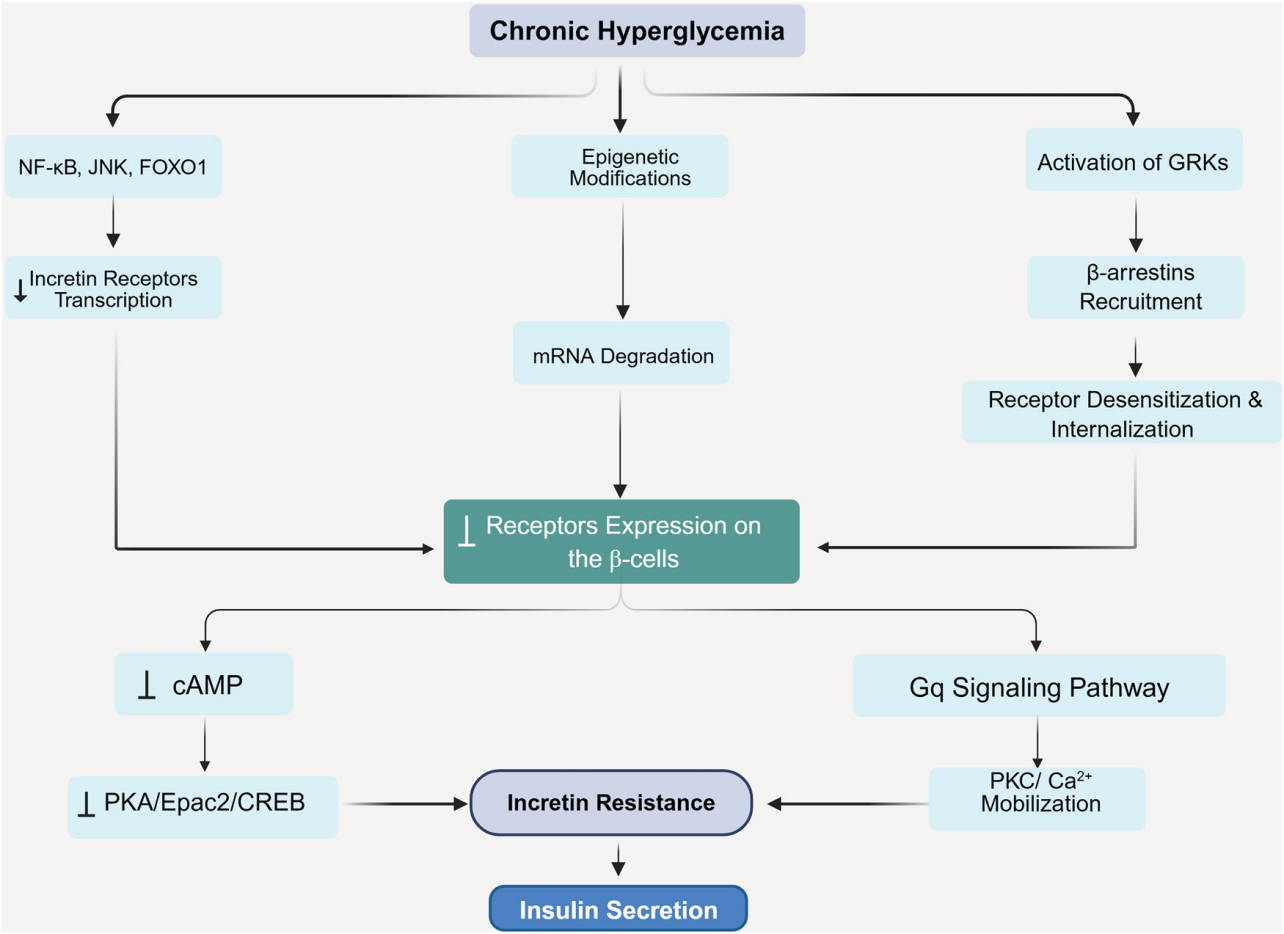
Dysfunction of intracellular signals in chronic hyperglycemia. This schematic illustrates the etiology of chronic hyperglycemia as a disruptor of intracellular signaling pathways, which subsequently impairs insulin secretion in pancreatic β-cells. Specifically, chronic hyperglycemia diminishes the activity of the cAMP/PKA signaling pathway, leading to a defect in cAMP/PKA signaling. This disruption results in elevated levels of ICER/PKA-Riα, thereby downregulating PKA activity. The consequent reduction in CREB phosphorylation suppresses insulin gene expression, terminating in decreased insulin secretion. Chronic hyperglycemia also induces downregulation of ARRB2 expression and impairs the ERK/CREB signaling pathways through biased signaling mechanisms. The diminished efficacy of these signaling pathways significantly contributes to the observed decline in insulin secretion. Furthermore, the effects of chronic hyperglycemia lead to a reduction in TCF7L2 levels, downregulating AKT signaling. Inhibition of insulin gene expression occurs as FOXO1 translocates into the nucleus due to the suppressed AKT pathway, whereby the inhibition of FOXO1 acts as a secondary factor, further exacerbating the decline in insulin secretion.

Desensitization of GPCRs to agonists may occur following prolonged stimulation of the receptors. In patients with T2DM, levels of GIP may remain stable or exhibit only slight decreases; however, obesity is associated with elevated postprandial GIP concentrations. The concept of homologous desensitization to GIP remains a theoretical construct and should not be postulated as the underlying hypothesis for the GIP resistance observed in individuals with T2DM. Theoretically, even maximal stimulation with GIP or GLP-1 that escalates insulin secretion may subject the receptor to desensitization due to G protein-coupled receptor kinases (GRKs) mediated phosphorylation of the receptor, recruitment of β-arrestins and internalization of the receptor, limiting receptor availability [32]. Moreover, hyperglycemia per se accelerates receptor turnover; elevated glucose in vitro stimulates GLP-1Rs internalization, thus enhancing their clearance from the cell surface [33]. In glucotoxicity conditions, this internalization is mediated by protein kinase A (PKA), which phosphorylates critical residues in the receptor to mark it for endocytosis [34]. Thus, there is an overall negative effect of T2DM at the receptor level mediated by a decrease in receptor expression, and for some of these remaining receptors, functional impairment is present, along with defective incretin signaling. However, despite the implementation of such restrictions, GLP-1 can enhance insulin secretion in many patients with T2DM by leveraging residual β-cell cAMP signaling and extrapancreatic mechanisms. Figure 1 highlights the downregulation and desensitization of incretin receptors.

### Impaired intracellular signaling pathways

#### cAMP/PKA signaling defect

GLP-1R and GIP-R couple to Gs proteins, which activate adenylyl cyclase (AC), leading to an increase in pancreatic β-cell intracellular cyclic AMP (cAMP) levels; this rise in cAMP promotes insulin secretion through the PKA and Epac2 pathways [35]. In the case of T2DM, post-receptor signaling is impaired, resulting in a diminished downstream response. Chronic hyperglycemia is a key contributor to this impairment, as it increases basal cAMP levels and upregulates feedback from upstream regulators that inhibit the cAMP-PKA axis [36]. For instance, under conditions of glucotoxicity, β-cells exhibit an upregulation of inducible cAMP early repressor (ICER) and the PKA regulatory subunit RIα, accompanied by decreased PKA activity. Consequently, glucose-stimulated insulin secretion (GSIS) and GLP-1-mediated phosphorylation of PKA substrates, including CREB, are both reduced in T2DM islets compared to those from non-diabetic individuals [32]. In T2DM, incretin signaling is reduced due to compensatory cellular mechanisms that inhibit the generation of cAMP or decrease downstream signaling through PKA. At a mechanistic level, chronic hyperglycemia and metabolic stress initiate negative feedback and counter-regulatory processes through the upregulation of phosphodiesterase enzymes, which degrade cAMP, and alterations in adenylate cyclase function, leading to a reduction in cAMP availability [37]. Thus, diminished cAMP/PKA signaling reduces the physiological action of incretins on insulin secretion and restrains the β-cell response under diabetogenic conditions [38]. In the end, incretin signal amplification is impaired in T2DM cases, as the incretin signal fails to sustain cAMP/PKA activity.

#### β-arrestin and biased signaling

In mice β-cells, ARRB2 typically limits GLP-1-induced cAMP elevation at normal hormone levels. Although a reduction in ARRB2 can enhance cAMP signaling within cAMP-dependent pathways, ARRB2 is necessary for GIP-induced insulin secretion. Consequently, decreased expression of ARRB2 in T2DM may lead to further impairment of GIP [39]. GLP-1 can engage extracellular signal-regulated kinases 1 and 2 (ERK1/2) and cAMP response element-binding protein (CREB) through a β-arrestin–dependent pathway; thus, alterations in ARRB2 may twist GLP-1 receptor signaling away from pro-survival pathways (i.e., ERK/CREB) [39]. This concept of biased agonism is increasingly recognized as relevant to the remodeling of incretin signaling in states of T2DM.

Beyond the extensively researched role of β-arrestin-2 in facilitating receptor desensitization and biased signaling of GPCRs, we propose that the cellular expression and functionality of β-arrestin-2 are imprinted in an epigenetically determined manner in response to early alterations in glycemic homeostasis, a phenomenon called metabolic memory. This imprinting may occur through mechanisms such as histone acetylation and the involvement of long non-coding RNAs at the ARRB2 locus, leading to long-term repression of β-arrestin-2 and attenuation of receptor desensitization. Additionally, we suggest that dysregulated signaling is continued through neuroendocrine feedback mechanisms, particularly those downstream of vagal afferent inputs and hypothalamic-pituitary-adrenal (HPA) axis signaling. This central-peripheral crosstalk may further modulate β-arrestin-2 activity in enteroendocrine and pancreatic β cells, resulting in a maladaptive gut-brain-β arrestin axis. We conclude that β-arrestin-2-mediated incretin resistance in T2DM arises from the conjunction of epigenetic downregulation and neuroendocrine enhancement of disrupted signaling pathways. This model provides a foundation for understanding individual variability in incretin response and identifies several therapeutic targets, including epigenetic modulators and neuromodulator interventions, which may possess the potential to reverse receptor desensitization and restore receptor signaling plasticity.

#### ERK1/2 and PI3K/AKT pathways

**Fig. 2:**
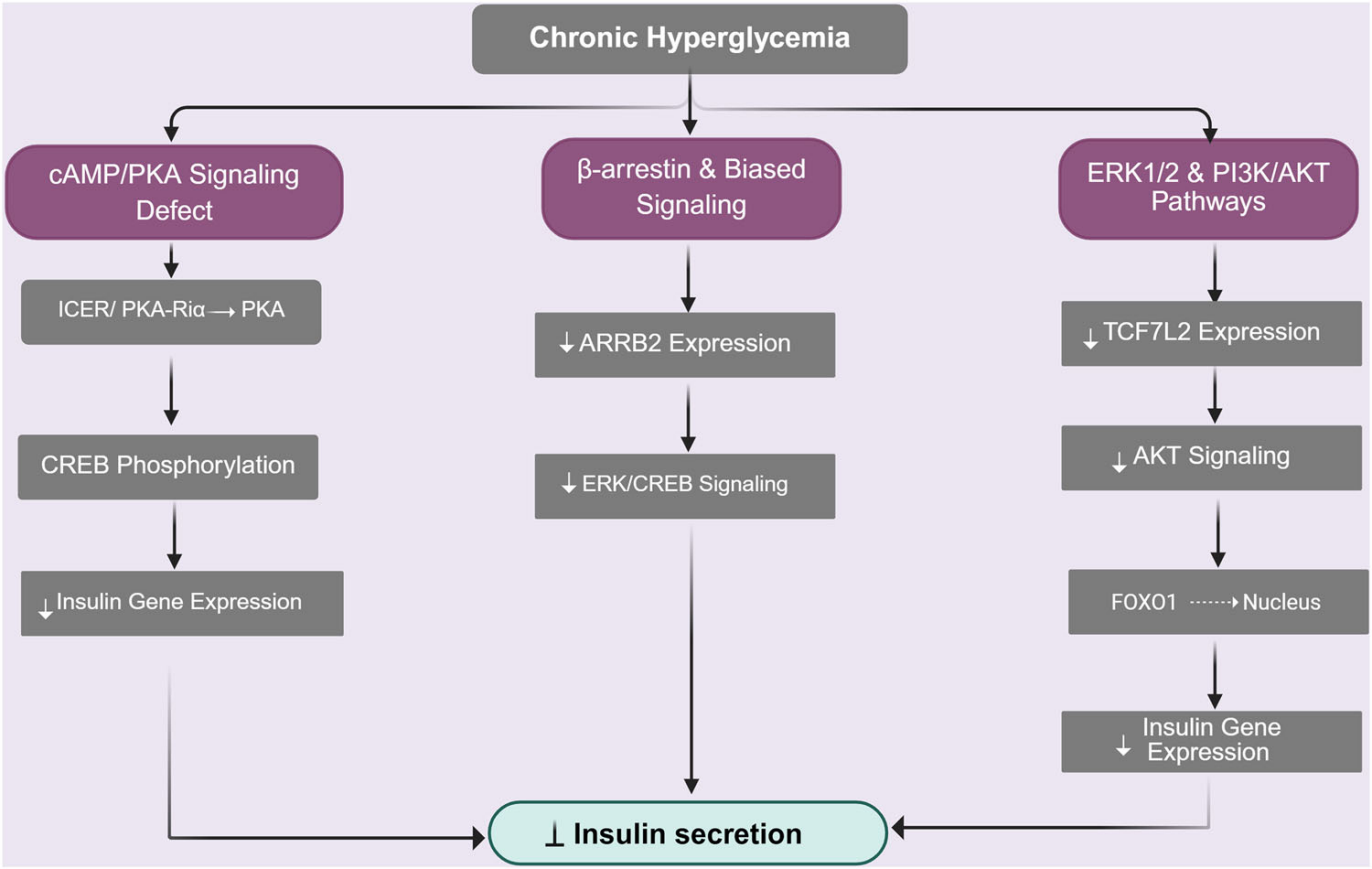
Chronic hyperglycemia-induced incretin resistance. This illustration elucidates the mechanisms by which chronic hyperglycemia induces incretin resistance, thereby impairing insulin secretion through alterations in receptor function and signaling cascades within β-cells. Under hyperglycemic conditions, the elevation of stress-responsive transcription factors such as NF-κB, JNK, and FOXO1 constrains the transcription of incretin receptors, reducing receptor availability. Furthermore, hyperglycemia induces epigenetic modifications that increase mRNA degradation, significantly diminishing incretin receptor expression on pancreatic β-cells. Chronic hyperglycemia also enhances the activity of G protein-coupled receptor kinases (GRKs) and promotes the recruitment of β-arrestins, mechanisms that diminish receptor expression at the cell surface by facilitating receptor desensitization and internalization. These mechanisms substantially reduce receptor expression, inhibit cAMP-mediated signaling (PKA/Epac2/CREB), and disrupt signal transduction through Gq pathways (PKC/Ca²⁺ mobilization). Such alterations may significantly contribute to the development of incretin resistance, leading to a marked reduction in insulin secretion.

Incretin receptors can activate additional signaling pathways beyond cAMP. GLP-1 functions by activating the β-cell phosphoinositide 3-kinase (PI3K)/AKT and mitogen-activated protein kinase (MAPK, specifically ERK1/2) pathways, which promote cell survival and the transcription of genes that facilitate cell proliferation [40]. In states of incretin resistance, there appears to be dysfunction within these signaling network pathways. Indeed, when human islets are “primed” for T2DM through the downregulation of the Wnt pathway transcription factor TCF7L2, both GLP-1 and GIP fail to stimulate further phosphorylation of AKT [41]. Consequently, downstream transcriptional factors such as FOXO1 become hypophosphorylated and sequestered in the nucleus, inhibiting the transcription of proinsulin genes. This extension of mitogenic and pro-survival signaling beyond acute insulin secretion suggests the phenomenon of incretin resistance. Further studies have demonstrated that GLP-1R coupling transitions from Gs to Gq proteins due to ER stress induction [42]. ER, stress in β-cells resulted in the activation of GLP-1 signaling via Gq, which mobilizes calcium ions and protein kinase C (PKC), rather than the conventional cAMP pathway [43]. Although Gq coupling may preserve some insulin secretion, it constitutes abnormal signaling relative to cAMP and can be viewed as a compensatory mechanism as the cell endeavors to compensate for dysfunction within the cAMP signaling pathway [44]. A reduction in β-arrestin signaling was found to increase the activation of the cAMP/protein kinase A (PKA) pathway but concurrently diminished the activation of the ERK pathway, which serves as a critical intersection for cAMP/PKA and PI3K/AKT dynamics, ultimately leading to impaired insulinotropic potency of incretins in T2DM [45, 46]. Therefore, the dysfunctional downstream signaling of all three pathways, cAMP/PKA, β-arrestin/ERK, and PI3K/AKT, compromises the efficacy of incretins in T2DM. Figure 2 represents a schematic summarizing the molecular mechanisms involved in incretin resistance mediated by the impairment of intracellular signaling pathways.

### Role of inflammation, oxidative stress, and ER stress

#### Islet inflammation

T2DM is characterized by chronic low-grade inflammation. Elevated levels of pro-inflammatory cytokines, such as interleukin-1β (IL-1β) and tumor necrosis factor-alpha (TNF-α), have been detected in the pancreatic islets of patients with T2DM, particularly in those exhibiting glucotoxicity. A specific example is IL-1β, which has been demonstrated to impair the function and viability of β-cells, inhibiting glucose-induced insulin secretion and inducing activation of nuclear factor kappa B (NF-κB) and apoptosis. Such an inflammatory environment diminishes the responsiveness of β-cells to incretins [47]. Inflammation is a potentiator of incretin resistance, contributing to β-cell dysfunction through toxic effects on the cells and altering their signaling pathways, resulting in a condition where all standard incretin signals yield less insulin release [48].

Glucotoxicity, both in vitro and in vivo, is functionally characterized by prolonged exposure to elevated concentrations of glucose. This condition is associated with impaired first-phase GSIS, elevated proinsulin-to-insulin ratios, and markers of cellular stress, which include the induction of CHOP, BiP/GRP78, splicing of XBP1, and the production of reactive oxygen species (ROS) [49].

#### Oxidative and ER stress

Oxidative stress occurs when ROS production exceeds the antioxidant capacity of cellular substances, such as glutathione, resulting in the disruption of ATP-dependent closure of K⁺ channels, alterations in voltage-gated Ca²⁺ influx, and ultimately the inhibition of granule exocytosis [50]. Moreover, oxidative stress activates stress-activated kinases, particularly c-Jun N-terminal kinase (JNK) and p38 MAPK, which further disrupt the insulin secretory cascade and may lead to β-cell apoptosis [51]. Pancreatic islets derived from human and rodent models of T2DM exhibit an increased accumulation of markers associated with oxidative stress and the UPR [52]. When protein is not bioavailable, these stress states can disrupt the biosynthesis of hormones and inhibit proper protein function. ER stress alters signaling pathways via the GLP-1 receptor, leading to a bias away from cAMP signaling [53].

Furthermore, ER stress can inhibit the biosynthesis and proper folding of incretin receptors, decreasing receptor availability on the cell membrane. Glucotoxicity accelerates the overproduction of ROS through enhanced mitochondrial electron transport, the activation of the polyol pathway, and the formation of advanced glycation end products (AGEs) [54]. GLP-1 agonist has been reported to inhibit oxidative stress markers, cytokine signaling, and apoptosis in pancreatic islets [55].

### Crosstalk with insulin resistance and β-cell dysfunction

#### Insulin resistance feeds incretin resistance

Peripheral insulin resistance, characterized by muscle, liver, and adipose tissue dysfunction, is a defining feature of T2DM and results in increased insulin secretion from pancreatic β-cells [56]. Chronic hyperglycemia, along with elevated levels of non-esterified fatty acids and cytokines, leads to the sustained activation of β-cell glucose and stress-sensing pathways, including mTORC1, oxidative stress responses, and the ER/UPR. This prolonged stimulation places considerable strain on the secretory capacity of β-cells [57]. Resistance to incretin hormones is exacerbated by markers of glucotoxicity, such as chronic hyperglycemia, which induce hormonal resistance primarily through mechanisms involving oxidative stress, ER stress, chronic inflammation, the AGEs, and the impairment of pancreatic β-cells [58]. The cumulative effects of these processes manifest as compromised GLP-1 and GIP receptor signaling, diminished receptor expression and sensitivity, and reduced insulinotropic responses. Elevated free fatty acids can also provoke lipotoxic stress within pancreatic islets, potentially impairing GIP and GLP-1 signaling pathways [59]. Notably, the reduction in incretin effect observed in individuals who subsequently develop insulin resistance precedes the onset of diabetes [60]. At a mechanistic level, the interplay of hyperinsulinemia and hyperglycemia within the context of insulin resistance may lead to the downregulation of incretin receptors and impaired GLP-1 secretion.

#### β-cell dysfunction limits incretin action

Incretins exert their effects primarily through pancreatic β-cells; thus, any reduction in β-cell mass or functionality adversely impacts the actions of incretin hormones [61]. These hormones only partially mitigate the progressive β-cell dysfunction observed in individuals with T2DM [62]. For example, individuals with advanced-stage T2DM and limited insulin reserves exhibit only modest responses to the most potent GLP-1 agonists [63]. The response to GLP-1 is particularly impaired in patients with prolonged disease duration or reduced β-cell reserves. When the healthy β-cell mass is diminished, the capacity for incretin-stimulated insulin secretion is consequently limited.

In contrast, therapies that restore β-cell function are generally associated with improved incretin sensitivity [46]. Specific non-incretin antidiabetic therapies have demonstrated the ability to enhance β-cell responsiveness to GLP-1 and GIP [64]. Intensive insulin therapy, SGLT2 inhibitors, and lifestyle modifications that lower blood glucose levels can restore the incretin effect, partially recovering β-cell function [58]. For instance, clamp studies indicated that insulin secretion in glucose-responsive T2DM patients improved after 2 weeks of treatment with the SGLT2 inhibitor dapagliflozin, demonstrating enhanced responses to GLP-1 and GIP due to the mitigation of hyperglycemia [65]. Therefore, a bidirectional interaction is suggested that insulin resistance and β-cell failure induce incretin resistance, while reducing metabolic stress on β-cells can partially restore incretin responsiveness.

#### The theory behind the inferior efficacy of GIP relative to GLP-1 in T2DM

Several mechanisms modulate GLP-1 function in relation to GIP: (i) The signaling pathway mediated by the GLP-1 receptor (GLP-1R), involving cAMP and EPAC2/PKA, remains active under stress conditions despite reduced availability of the GIP receptor (GIPR); (ii) β-arrestin 2, a protein whose levels may decline with reduced β-cell reserve and failure, plays a more critical role in GIP activity than in GLP-1 activity [39]; (iii) Endoplasmic reticulum (ER) stress can modify GLP-1R signaling, facilitating partial engagement of Gq and the preservation of Ca²⁺ mobilization [43]; and (iv) GLP-1 exerts extrapancreatic effects, such as delaying gastric emptying and suppressing glucagon secretion, which contribute to glycemic control in conditions of low insulin reserve [4]. Furthermore, significant differences in receptor trafficking and reserve between GLP-1R and GIPR limit ligand access to GIPR compared to the rarer α-cells [66]. This leads to early-onset partial GIP resistance while maintaining sustained responsiveness to GLP-1.

### Genetic and epigenetic contributors

#### TCF7L2 and incretin resistance

Polymorphisms in the TCF7L2 gene are associated with the risk of T2DM; however, their association with altered incretin responses appears limited [67]. Moreover, there is insufficient evidence to suggest that common genetic variations within this gene contribute to prevalent forms of incretin resistance in diabetes. Notably, associations with other polymorphisms are infrequent [68]. On the other hand, it is noteworthy that variants of TCF7L2 are associated explicitly with intolerance to incretin action [69]. Carriers of the risk allele, such as the TCF7L2 rs7903146 T allele, exhibit normal or even elevated levels of incretins [70]; however, they demonstrate a poor response to incretins in relation to insulin secretion. In non-diabetic obese humans, this risk variant has been correlated with a diminished incretin effect, characterized by elevated glucose levels during an oral glucose tolerance test despite higher plasma levels of GLP-1, indicating an impaired ability of β-cells to respond to GLP-1. At the molecular level, TCF7L2 appears to modulate the components of the incretin pathway within pancreatic β-cells. Evidence of TCF7L2 function in humans is derived from observations of reduced TCF7L2 protein levels in pancreatic islets of individuals with T2DM despite upregulation of mRNA levels [71]. Experimental knockdown of TCF7L2 in human islets results in downregulation of GLP-1R and GIP-R; this knockdown also impairs insulin secretion in response to GLP-1 or GIP stimulation, although basal and non-incretin-stimulated secretion remains intact [72]. These findings suggest that TCF7L2 may serve as a critical link between genetic risk and incretin resistance; loss of function in TCF7L2 disrupts the maintenance and signaling of incretin receptors, leading to β-cell failure during T2DM.

Furthermore, other gene variants outside of TCF7L2 may modulate the incretin response. A significant loss-of-function variant of the human GIPR (E354Q) has been identified, which phenocopies a GIP-resistant state [73]. KCNQ1 has also been recognized as a susceptibility gene for T2DM, expressed in enteroendocrine cells and β-cells.

These genetic findings highlight that incretin resistance possesses a heritable component, suggesting that some individuals may have β-cells that are inherently less sensitive to incretins or that their gut cells secrete lower levels of hormones compared to individuals without the expressed variants. However, we propose that individual variation in incretin response is not only shaped by genetic architecture but also by circadian timing and sex, which may also impact individual responses to incretin hormones. The effect of circadian and sex on incretin pharmacodynamics holds plausible biological significance; however, these factors remain insufficiently explored. Current evidence from animal studies is limited, and the data from human studies are notably lacking. Moreover, males and females use different mechanisms to control specific genes, especially those found on only the X chromosome, explaining some of the different responses to treatment observed in patients. Together, these ideas endorse a more individualized approach to incretin therapy. Moreover, males and females also show sex-specific differences in regulating specific genes, such as some located on the X chromosome, which may partially translate into differences in treatment response among patients. All of these concepts are collectively in favour of a more patient-tailored approach to incretin therapy. In the majority of patients, genetics is now regarded as a modifier of incretin biology rather than a primary etiological factor.

#### Epigenetic and developmental influences

Glucotoxicity may contribute to gene silencing through epigenetic modifications, such as promoter methylation of GLP1R or GIPR or downregulation of transcription factors like PDX1 that mediate incretin actions [74, 75]. A human pancreatic islet epigenome-wide study has identified differential DNA methylation associated with T2DM, involving insulin secretion and GPCR signaling pathways [76]. Epigenetic dysregulation may contribute to incretin resistance and explain why it can develop independently of changes in DNA sequence. An abnormal maternal metabolic environment during pregnancy induces permanent epigenetic alterations of the incretin axis, leading to persistent functional disorders of incretin hormones and an increased risk of diabetes later in life [77]. Glucotoxicity and chronic inflammation induce not only epigenetic modifications involving DNA methylation and histone modification, which are significant mechanisms regulating incretin receptor genes, but also higher-order regulatory mechanisms that ensure the stability of these gene-silencing epigenetic states. These mechanisms include alterations to chromatin architecture, such as enhancer-promoter decoupling, the repositioning of genomic loci into repressive nuclear compartments, and the engagement of long non-coding RNAs that scaffold transcriptional repressors. These features may represent a form of metabolic memory whereby incretin resistance persists even after restoring glycemic control. We propose that such persistent epigenetic silence may be reversible through targeted chromatin topology and RNA-mediated repression modulation. Locus-specific strategies for restoring incretin receptor expression may include CRISPR-dCas9-based epigenetic editors and lncRNA-directed antisense oligonucleotides. Additionally, microbiota-accessible metabolites could influence host histone-modifying enzymes, establishing a microbiome-epigenome-incretin axis. This integrated model provides a novel discovery platform for precision therapies aimed at reprogramming epigenetic barriers to incretin responsiveness.

#### Hypothesis of adaptive microbiota endocrine feedback

Microbial metabolites, particularly SCFAs such as acetate, propionate, and butyrate, are increasingly recognized as significant mediators of host-microbe communication through epigenetic regulation. SCFAs serve as endogenous inhibitors of histone deacetylases (HDACs) and influence DNA methylation and chromatin structure, thereby affecting transcriptional activity across a diverse array of cell types [78]. Given these well-characterized epigenetic mechanisms, we hypothesize that SCFAs may exert similar regulatory effects in enteroendocrine L-cells and pancreatic β-cells by inducing modifications in DNA methylation and histone acetylation, which could lead to enduring changes in gene expression. Furthermore, the stimulation of GLP-1 secretion in response to transient hypermetabolic conditions may induce shifts in gut microbiota composition to meet energy demands. This suggests that such epigenetic reprogramming could produce lasting alterations in circulating GLP-1 levels or receptor sensitivity, thereby linking microbial metabolism to host glycemic regulation.

On the other hand, recent studies suggest that circadian oscillations in the composition of the gut microbiome may regulate the rhythmic secretion of GLP-1, thereby aligning incretin signaling with the host’s metabolic rhythms [79]. Disruption of the cycling of microbial signals abolishes diurnal GLP-1 oscillations and impairs glucose homeostasis in murine models, thereby demonstrating a reciprocally responsive relationship between gut microbiota-derived signals and host endocrine rhythms [79]. Furthermore, small RNAs and bioactive proteins found in bacterial extracellular vesicles have been implicated in cross-kingdom communication that influences intestinal immunity and the expression of GLP-1 [80]. Collectively, the findings presented above may support the hypothesis that circadian dynamics in microbiota and vesicle-mediated communication both may play roles in incretin physiology and may contribute to interindividual variability in metabolic resilience and incretin mimetic drug response.

## Experimental models of incretin resistance

### In vitro studies

The role of incretins in β-cells can be studied under well-controlled conditions using immortalized insulin-secreting cell lines (such as INS-1 and MIN6 β-cells) or isolated pancreatic islets [81]. By subjecting these cells to a diabetic environment characterized by excess glucose, elevated lipid levels, or inflammatory cytokines, researchers can replicate aspects of T2DM and observe subsequent decreases in incretin receptor expression and signal transduction. For instance, repeated administration of GLP-1 to islets in vitro in the presence of chronic hyperglycemia has been shown to diminish GLP-1 receptor (GLP-1R) expression [82] and reduce cAMP production in response to GLP-1 [83]. Employing pharmacological inhibitors and molecular techniques (such as small interfering RNA (siRNA) or gene overexpression), these models have elucidated critical nodes involved in the process of GPCR signaling [84, 85]. For example, protein kinase A (PKA) inhibition has been demonstrated to block GLP-1R internalization in a high-glucose environment [32], while dominant-negative protein kinase C alpha (PKCα) can inhibit receptor downregulation via hyperglycemic pathways [86]. These findings light our understanding of the mechanisms underlying incretin resistance due to glucotoxicity.

### Animal studies

Rodent models of T2DM are extensively utilized for studying incretin defects [87]. Commonly employed models include those induced by high-fat diets, as well as genetic models such as leptin-deficient ob/ob mice and obese db/db mice, in addition to rat models like the Zucker diabetic fatty rat. These animals exhibit a diminished incretin effect that parallels the condition observed in human T2DM; notably, diabetic rats display no insulinotropic response to GIP. Researchers extrapolate data regarding incretin hormone levels, receptor expression, and insulin secretion, with or without interventions, utilizing these models. The 90% pancreatectomized rat develops chronic hyperglycemia and simulates changes in human islets [88], including alterations in incretin receptors. Animal studies have demonstrated that phlorizin normalizes blood glucose levels and can restore incretin receptor levels [89]. Genetic knockout models further enhance our understanding; for instance, studies involving GLP-1 receptor knockout (GLP-1R⁻/⁻) and GIP receptor knockout (GIPR⁻/⁻) mice have elucidated the specific contributions of each peptide to glucose homeostasis [90]. These models are also employed to evaluate investigational therapies, such as GIP receptor antagonists or dual agonists [91], to assess their efficacy in overcoming incretin resistance. The limitations of models such as Non-NMINS 1/MIN6 and various transgenic or rodent models are evident in their limited capacity to replicate the characteristics of T2DM [92]. Notably, these models demonstrate significant divergence from human β-cell physiology, particularly in terms of NADPH utilization, ion channel expression, and the density and trafficking of incretin receptors [93]. Consequently, these findings underscore the necessity for validation using human islets and, when appropriate, in vivo physiological studies to mitigate the risks of over-extrapolation.

### Clinical studies

The study of incretin function is conducted using various clinical research techniques. One gold-standard approach involves the hyperglycemic clamp with incretin infusion: investigators maintain a fixed high blood glucose level and infuse GLP-1 or GIP, thereby quantifying β-cell responsiveness [94]. Patients with T2DM exhibit significantly decreased insulin responses to GLP-1/GIP infusions compared to subjects without diabetes, which enables the quantification of incretin resistance in T2DM patients [95]. Another method employs an oral versus intravenous glucose tolerance test to measure the incretin effect; patients with T2DM demonstrate an incretin effect approaching zero, as equivalent insulin profiles are recorded following oral and intravenous glucose administration [96]. To investigate molecular expression, isolated islets from visceral organ donors with T2DM were used to measure signaling protein levels in T2DM versus non-diabetic (ND) islets [97], such as GLP-1 receptor (GLP-1R) levels. Novel approaches, including single-cell RNA sequencing of islets, are beginning to identify subpopulations of cells with unusual incretin signaling in T2DM [98].

Regarding incretin secretion, the mild GLP-1 secretory defect has been elucidated by measuring GLP-1/GIP release during meal tests in T2DM patients compared to controls, who are well-matched for obesity [90]. Ex vivo gut models and enteroendocrine cell lines are also being utilized to evaluate whether natively present diabetic conditions alone impact GLP-1/GIP secretion at the source [99]. These models are designed to determine if incretin sensitivity can be reversed in specific treatment options for patients taking SGLT2 inhibitors, statins, and anti-inflammatory drugs by measuring the incretin effect before and after therapy.

## Impact of understanding incretin resistance on current incretin-based therapies

Incretin-based therapies, particularly GLP-1RAs and DPP-4 inhibitors, have become foundational components of pharmacologic treatment for T2DM. GLP-1RAs exhibit glucose-dependent insulinotropic effects, associated with significant weight loss and indirect cardiovascular benefits [100]. In contrast, DPP-4 inhibitors are considered weight-neutral [101], with no evidence of cardiovascular risk reduction observed in human studies [102]. Thus, each class of incretins mimics should be evaluated separately in clinical practice. Compared to traditional therapies, including sulfonylureas and insulin, GLP-1RAs have a lower risk of hypoglycemia and provide advantages for patients who need to manage their weight. However, the effectiveness of these agents can be limited by incretin resistance, which includes impaired GLP-1 and GIP signaling at various levels, such as hormone secretion, receptor expression, and intracellular signaling pathways [103].

In contrast to insulin therapy, which facilitates glucose uptake regardless of β-cell function, incretin-based agents depend on the responsiveness of β-cells and, consequently, demonstrate reduced efficacy during the later stages of the disease. Conversely, sodium-glucose cotransporter 2 (SGLT2) inhibitors function independently of insulin signaling, thereby preserving their effectiveness as β-cell function deteriorates and thus proves advantageous across various stages of the disease. Beyond the β cell, GLP-1 receptor agonists inhibit glucagon secretion, slow gastric emptying, and facilitate weight loss, mechanisms that substantially enhance glycemic control and metabolic outcomes in individuals with T2DM [104]. Hence, the therapeutic benefits of incretin therapies are most pronounced in individuals with early to moderate T2DM, with their efficacy diminishing in the context of ongoing metabolic stress and β-cell failure [105]. To date, attempts to develop strategies targeting GIPR have encountered limited success, primarily due to GIPR resistance and insufficient insulin-stimulating effects in T2DM patients. However, recent pharmacological advancements have yielded promising results, particularly the emergence of dual GLP-1/GIP receptor agonists such as tirzepatide. Direct comparisons between the dual GLP-1/GIP agonist tirzepatide and semaglutide, administered at a dosage of 1.0 mg once weekly, reveal superior effects on glycemic control and weight loss, as evidenced by publicly available data against a glucagon analogue [106]. These discrepancies may be attributed to variations in dosing, drug exposure, and the degree of activation of the murine GLP-1 receptor (GLP-1R) [94]. Furthermore, the role of GIP agonism in these effects in humans remains a topic of ongoing debate. Notably, it has yet to be demonstrated that exogenous GIP induces weight loss; genetic studies indicate that loss-of-function mutations in the GIP receptor (GIPR) are associated with leanness, suggesting a complex interplay of factors. Consequently, the superior efficacy of tirzepatide does not necessarily support the assertion that GIP reverses β-cell responsiveness to glucose. A comprehensive understanding of the molecular events contributing to incretin resistance can facilitate more personalized pharmacotherapy for T2DM. Generally, patients exhibiting significant β-cell impairment or low GLP-1 receptor expression, evidenced by a poor response to GLP-1 receptor agonists or confirmed through molecular profiling, may derive more significant benefits from strategies that directly address metabolic burdens, such as sodium-glucose co-transporter 2 (SGLT2) inhibitors or combinations with tirzepatide. Given the mechanisms underlying signaling abnormalities, including receptor downregulation resulting from inflammation, oxidative stress, and ER stress, it is plausible that adjunctive anti-inflammatory or antioxidant therapies could further enhance incretin sensitivity [107]. Moreover, the β-arrestin pathway-mediated desensitization of receptor signaling and the persistent epigenetic repression of receptor gene expression suggest potential therapeutic roles for targeted epigenetic therapies or chronopharmacological strategies to restore β-cell functionality. For patients experiencing high metabolic stress, temporarily withdrawing incretin therapies and transitioning to agents that stabilize glycemia may enhance subsequent responsiveness, underscoring the significance of therapy sequencing and cycling influenced by the disease’s physiology. Ultimately, the therapeutic efficacy of incretin-based agents is contingent upon the degree of underlying resistance. While all GLP-1 receptor agonists (GLP-1RAs) uniformly facilitate weight reduction and improve glycemia, their effects are diminished in individuals with low β-cell mass or chronic hyperglycemia, conditions characterized by persistent receptor internalization and signaling failure. This manuscript seeks to elucidate how oxidative and ER stress disrupt GLP-1 signaling, negating the effects of even high-dose agonists. Similarly, GIP activity in T2DM has significantly limited its clinical applicability; however, this limitation may be alleviated through pathway coactivation, as suggested by recent data on co-agonist therapeutics [66].

Interventions aimed at mitigating glucotoxicity, including short-term intensive insulin therapy, SGLT2 inhibition, and weight reduction, have produced only modest improvements in incretin responses. Nevertheless, the efficacy of these strategies seems limited, raising questions about their clinical relevance in the setting of advanced disease stage of T2DM. This approach improves therapeutic efficacy and aligns with the broader trend toward personalized, mechanism-guided diabetes treatment.

## Conclusion

Incretin resistance in T2DM should be regarded as a relative and context-dependent phenotype that reflects underlying beta cell deficits, including the loss of first-phase insulin secretion, oxidative ER stress, and transcriptional reprogramming, particularly under conditions of systemic metabolic stress, rather than merely an abnormality at the receptor level. Current human data do not support the latter aspect of desensitization theory, indicating that receptor disturbances account for only a minor fraction of the observed desensitization. Incretin receptor responses have significant clinical implications, as they not only stimulate β-cells but also inhibit glucagon secretion, enhance gastric emptying, and promote weight reduction. Nevertheless, the primary defect remains the diabetic β cell’s inability to produce and release insulin. Thus, mechanistic approaches aimed at mitigating glucotoxicity, lipotoxicity, and inflammation, as well as restoring β-cell function, are crucial for realizing the full therapeutic potential of incretin-based interventions. Potential mechanisms, including the roles of the microbiome, circadian rhythms, epigenetic remodeling, warrant investigation; however, they have yet to be validated in human studies.
